# Programmable half-life and anti-tumour effects of bispecific T-cell engager-albumin fusions with tuned FcRn affinity

**DOI:** 10.1038/s42003-021-01790-2

**Published:** 2021-03-08

**Authors:** Ole A. Mandrup, Sui Ching Ong, Simon Lykkemark, Anders Dinesen, Imke Rudnik-Jansen, Niels Frederik Dagnæs-Hansen, Jan Terje Andersen, Luis Alvarez-Vallina, Kenneth A. Howard

**Affiliations:** 1 Interdisciplinary Nanoscience Center (iNANO), Department of Molecular Biology and Genetics, Aarhus University, Aarhus C, Denmark; 2 Department of Biomedicine, Aarhus University, Aarhus C, Denmark; 3 Department of Immunology, University of Oslo, Oslo University Hospital Rikshospitalet, Oslo, Norway; 4 Institute of Clinical Medicine and Department of Pharmacology, University of Oslo, Oslo, Norway; 5 Cancer Immunotherapy Unit (UNICA), Department of Immunology, Hospital Universitario 12 de Octubre, Madrid, Spain; 6 Immuno-Oncology and Immunotherapy Group, Instituto de Investigación Sanitaria 12 de Octubre (i + mas12), Madrid, Spain

**Keywords:** Recombinant protein therapy, Cancer immunotherapy, Cancer immunotherapy

## Abstract

Fc-less bispecific T-cell engagers have reached the immuno-oncology market but necessitate continual infusion due to rapid clearance from the circulation. This work introduces a programmable serum half-life extension platform based on fusion of human albumin sequences engineered with either null (NB), wild type (WT) or high binding (HB) FcRn affinity combined with a bispecific T-cell engager. We demonstrate in a humanised FcRn/albumin double transgenic mouse model (AlbuMus) the ability to tune half-life based on the albumin sequence fused with a BiTE-like bispecific (anti-EGFR nanobody x anti-CD3 scFv) light T-cell engager (LiTE) construct [(t_½_ 0.6 h (Fc-less LiTE), t_½_ 19 hours (Albu-LiTE-NB), t_½_ 26 hours (Albu-LiTE-WT), t_½_ 37 hours (Albu-LiTE-HB)]. We show in vitro cognate target engagement, T-cell activation and discrimination in cellular cytotoxicity dependent on EGFR expression levels. Furthermore, greater growth inhibition of EGFR-positive *BRAF* mutated tumours was measured following a single dose of Albu-LiTE-HB construct compared to the Fc-less LiTE format and a full-length anti-EGFR monoclonal antibody in a new AlbuMus *RAG1* knockout model introduced in this work. Programmable half-life extension facilitated by this albumin platform potentially offers long-lasting effects, better patient compliance and a method to tailor pharmacokinetics to maximise therapeutic efficacy and safety of immuno-oncology targeted biologics.

## Abstract (web summary)

Mandrup et al. describe a panel of recombinant albumin fusions, engineered with different affinities to the human neonatal Fc receptor to program the half-life extension of a bispecific (EGFR/CD3) T-cell engager. They show that this approach generates target engagement, T-cell activation, tunable in vivo half-life extension, cellular cytotoxicity dependent on the cell surface levels of EGFR and can inhibit growth of BRAF mutated EGFR-positive tumours in mice.

## Introduction

Cancer is a major cause of death with more than 1.8 million new cases estimated to be diagnosed in 2020 in the United States alone^1^. Cancer immunotherapy has risen from peripheral use to a front-line clinical treatment in just over a decade with several immuno-oncology drugs approved e.g. Keytruda^®^, Tecentriq^®^ and Blincyto^®^, and more than 1500 clinical trials currently active or recruiting in this area (ClinicalTrials.gov). In the immuno-oncology space, T-cell engaging bispecific antibodies are attracting considerable attention with a myriad of different engineered formats developed^2^. The excitement, in part, fuelled by the impressive response rates shown with blinatumomab (Blincyto^®^) in non-Hodgkin lymphoma^3^ and acute lymphoblastic leukaemia^4^ patients. Blincyto^®^ is a ~55 kDa bispecific T-cell engager (BiTE^®^) composed of two single-chain Fv (scFv) antibody fragments (anti-CD19 and anti-CD3) connected by a short flexible linker that mediates immunological synapse formation between CD19-positive cells and CD3-positive T-cells resulting in T-cell-mediated cell killing. Early studies with the T-cell binding OKT3 anti-CD3 antibody have, however, reported overzealous immune stimulation such as non-specific systemic cytokine release through binding of the Fc region to immune cell expressing Fc gamma receptors (FcɣR)^5,6^. More recently, a phase 1 clinical trial with the Fc containing bispecific anti-EpCAM x anti-CD3 antibody catumaxomab (Removab) was terminated following a patient fatality, underlining the potential severity of Fc-enhanced immune stimulation^7^ and requirement to mitigate adverse effects such as cytokine release syndrome and close monitoring of clinical trials with immune-stimulatory drugs^8,9^.

Removal of the Fc region is a standard strategy to circumvent adverse immune effects, but with a caveat of removing Fc-driven cellular recycling through engagement with the neonatal Fc receptor (FcRn) that results in drastic reduction in blood residence time. This is exemplified by the requirement of continuous intravenous (i.v.) infusion of Blincyto^®^ for up to 4 weeks per cycle of treatment^10^. Silencing Fc regions by removal of FcɣR binding by site-specific mutations is an approach to retain FcRn-driven half-life extension while reducing adverse immune induction. This, however, is challenging due to multiple different FcɣR and residual binding affinity shown in “silenced” Fc regions^11^. The number of mutations needed for silencing may further affect the stability and immunogenicity of the engineered Fc regions^12^. Several studies have shown that fusion to albumin-binding peptides^13^ or protein^14–16^ domains can successfully extend the half-life of proteins and antibody fragments. Half-life extension of nanobodies by a fusion to an albumin-binding nanobody in different species have been described by Hoefman et al.^17^ and one drug exploiting an albumin-binding nanobody (ALX-0061) has reached phase IIb clinical trial (ClinicalTrials.gov). Direct fusion of drugs to recombinant human serum albumin (HSA), however, offers the opportunity to control and fine-tune the half-life of fusion constructs through engineered FcRn binding affinities. HSA has a circulatory half-life of ~19 days that is predominately facilitated by engagement with FcRn^18^ but without FcɣR and C1q binding associated with IgG-derived Fc fragments^19^. Inspection of the binding interface of albumin with FcRn^20,21^ has allowed design of recombinant variants with different FcRn affinities by single-point amino acid mutations that have been subsequently used to tune the pharmacokinetic (PK) profile of albumin fusions^22,23^. Inclusion of human albumin sequences engineered with different FcRn affinities into BiTE-like designs offers molecules displaying different PK that could be a crucial parameter to select molecules with maximum efficacy and low side-effects. Bispecific antibodies may also provide an effective treatment for solid tumours non-respondent to available monoclonal antibodies such as the commercial anti-epidermal growth factor receptor (EGFR) antibody cetuximab that shows no efficacy as a monotherapy in *BRAF*-mutated colorectal cancer^24^. This may be mitigated using a bispecific anti-EGFR x anti-CD3 utilising the EGF receptor for induction of T-cell-mediated cell killing^25^. A recent example of this strategy is the bispecific “light” T-cell engager (LiTE) antibody comprised of an anti-EGFR nanobody fused with an anti-CD3 scFv that exhibits T-cell-induced killing of EGFR-positive cancer cells^26^.

**Fig. 1:**
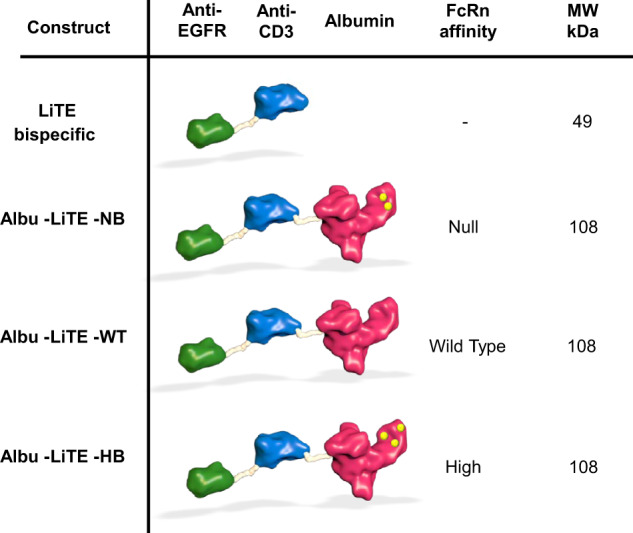
Schematic overview of designed Albu-LiTE constructs. The bispecific LiTE molecule (49 kDa) containing an anti-EGFR nanobody (green) linked to anti-CD3 scFv (blue). HSA (red) and point mutationed variants (yellow dots) with different affinities for human FcRn were genetically fused with the LiTE and expressed as a single polypeptide (108 kDa).

Here we report on a molecular concept where LiTE is genetically fused to HSA variants engineered with different human FcRn affinity for programmable PK. Proof-of-concept is provided in a human FcRn and human albumin double transgenic mouse model (AlbuMus)^27^ with bispecific anti-EGFR x anti-CD3 LiTE fusions (Albu-LiTE) engineered for null (Albu-LiTE-NB), wild-type (Albu-LiTE-WT) or high (Albu-LiTE-HB) FcRn binding (Fig. 1). Furthermore, in a novel AlbuMus *RAG1* knockout model (AlbuMus *RAG1* KO) bearing EGFR-positive *BRAF* mutated tumours, we demonstrate superior anti-tumour effects after a single dose of the Albu-LiTE construct compared to a conventional Fc-less LiTE format and a full-length anti-EGFR monoclonal antibody.

## Results

The Albu-LiTE vector constructs were designed based on a previously described anti-EGFR x anti-CD3 bispecific antibody^26^ and genetically fused to the N-terminus of WT HSA or variants mutated for null binding (NB) or increased FcRn binding (HB). Transient transfection of HEK293E cells cultured in serum-free media yielded secreted proteins which were efficiently purified on an anti-albumin affinity column. Obtained Albu-LiTE proteins were analysed by Coomassie staining and Western blotting (Supplementary Fig. 1), and showed high purity and expected molecular weights with only minor amount of unfused albumin present after purification. Analytical size exclusion chromatography was run to further validate the size and monomeric state of the fusion proteins (Supplementary Fig. 2).

**Fig. 2:**
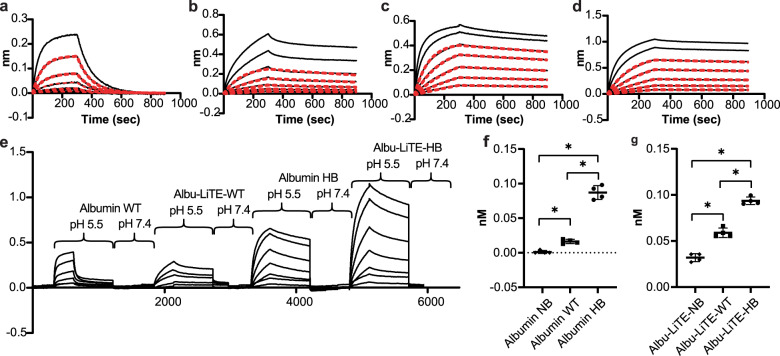
Kinetic measurements and cellular recycling. Sensorgrams showing binding of serial dilutions of protein to immobilised human FcRn at pH 5.5. **a** Binding curves for wild type human serum albumin (Albumin WT). **b** Binding curves for Albu-LiTE-WT. **c** Binding curves for high-binding HSA (Albumin HB). **d** Binding curves for Albu-LiTE-HB. The sensorgrams were fitted to a 1:1 model shown in red lines. **e** Bio-Layer Interferometry binding profile showing binding between FcRn and albumin at pH 5.5 mimicking the pH of late endosomes and at physiological pH 7.4. **f** Variant-dependent detection of recombinant albumin in supernatant from human FcRn expressing cells incubated with null binding HSA variant (triangle), WT HSA (square) and HB HSA variant (circle). **g** Variant-dependent detection of Albu-LiTE fusion protein in supernatant from cells incubated with Albu-LiTE-NB construct (triangle), Albu-LiTE-WT construct (square) and Albu-LiTE-HB construct (circle). Statistical analysis was made in GraphPad prism v.8 using a Mann–Whitney U-test, *p < 0.05.

Purified Albu-LiTE constructs were tested for binding to human FcRn at pH 5.5 using Bio-Layer Interferometry. The samples were compared to unfused WT recombinant HSA, a null binding HSA variant (NB) and a high affinity HSA variant (HB). The Albu-LiTE-WT and Albu-LiTE-HB constructs gave a good fit to a 1:1 binding model, comparable to the unfused WT and HB HSA variant (Fig. 2a–d). The NB construct resulted in a weak signal that could not be fitted, while the LiTE molecule resulted in a weak unspecific binding to FcRn at low pH which was also observed for the Albu-LiTE-NB; however, neither could be fitted with a 1:1 binding model (Supplementary Fig. 3). The sensorgram data showed a clear pH-dependent binding between the HSA component and human FcRn, with binding to the receptor at pH 5.5 and release after transfer to pH 7.4 (Fig. 2e). The estimated affinities after curve fitting indicated some influence from the bispecific fusion protein, but a clear difference between albumin sequences were still noticeable (Table 1).

**Table 1:** Human FcRn binding affinities for recombinant human serum albumin (HSA) variants and Albu-LiTE constructs.

Construct	*K*_D_ (M)	*k*_on_ (1/Ms)	*k*_dis_ (1/s)	*R*^2^ 1:1 fit	*χ*^2^ 1:1 fit
NB HSA	–	–	–	n.s.	–
Albu-LiTE-NB	–	–	–	n.s.	–
WT HSA	7.5E-07	1.3E+04	9.7E-03	0.995	0.029
Albu-LiTE-WT	1.1E-08	4.1E+04	4.7E-04	0.993	0.161
HB HSA	5.8E-09	4.0E+04	2.3E-04	0.999	0.058
Albu-LiTE-HB	2.9E-09	3.6E+04	1.0E-04	0.999	0.073

Measured binding affinities (*K*_D_, *k*_on_ and *k*_dis_) for HSA and Albu-LiTE constructs with *χ*^2^ and *R*^2^ for the fits are shown for each construct. Binding affinities could not be determined for the Null binding (NB) HSA and Albu-LiTE-NB construct due to low binding responses and lack of fit in a 1:1 binding model, *n.s.* non-significant.

To demonstrate human FcRn-mediated rescue from intracellular degradation by recycling in a cellular system, we utilised a recently established recycling assay^18,28^ where we compared the unfused HSA variants with that of the Albu-LiTE counterparts (Fig. 2f, g).

The results showed that the fusion design allowed the albumin to engage cellular expressed human FcRn and was recycled, thereby avoiding intracellular degradation. For the unfused HSA variants, 5-fold more of the HB was released back into the medium compared with the WT, while the NB was not rescued. The same trend was measured for the Albu-LiTE constructs, despite that more of the constructs were detected in the medium, possibly due to anti-EGFR nanobody in the LiTE construct binding to EGFR on the cell surface of the HMEC-1 cells (Supplementary Fig. 4). Increased recycling was comparable with that measured for the unfused HSA variant counterparts (Fig. 2f, g).

**Fig. 3:**
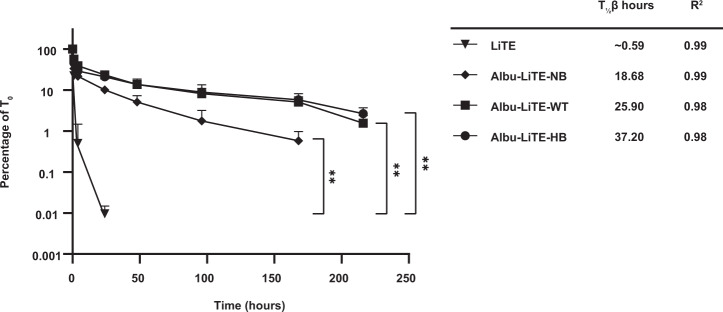
Pharmacokinetic analysis of LiTE and Albu-LiTE constructs in the AlbuMus model. Protein was i.v. injected in 4 groups of mice (*N* = 7 per group) and blood samples were drawn at 8 time points. Full size construct was detected in serum by ELISA. The *β* half-life and the *R*^2^ of the fit to the curve for each construct are listed for LiTE (triangle), Albu-LiTE-NB (diamond), Albu-LiTE-WT (square) and Albu-LiTE-HB (circle). Data was analysed with Graphpad prism v.8 using two-phase decay for calculating the molecular half-life (*T*_½_ *β*) and statistical differences between the constructs, detected in serum over time using a two-way ANOVA with multiple comparisons and Tukey’s post hoc correction. **p < 0.01. Error bars represent standard deviations.

To study the PK of the constructs in the double transgene (*hFcRn*^+/+^, *hAlb*^+/+^) AlbuMus model, the designed proteins were i.v. injected into the tail vein and blood samples drawn for analysis using an established ELISA that specifically capture the N-terminal nanobody and detects the C-terminal HSA for Albu-LiTE constructs or the C-terminal His-tag for the LiTE. Samples from each group (*N* = 7) were measured in triplicates and analysed using a two-phase decay model (Fig. 3).

The LiTE was rapidly cleared and not detectable beyond 24 h. When fused to HSA engineered for lack of human FcRn (Albu-LiTE-NB), the half-life was extended and detected up to 168 h that likely reflects increased molecular size. Further extended half-life was measured for Albu-LiTE-WT and Albu-LiTE-HB due to human FcRn-driven cellular recycling. The *β* half-lives derived demonstrated the longest half-life for Albu-LiTE-HB (*t*_½_ 37.2 h), which was more than 60-fold longer than that of LiTE (*t*_½_ 0.59 h), and 2-fold longer than that of the NB-containing LiTE variant (*t*_½_ 18.68 h). Compared to the Albu-LiTE-WT, the HB construct engineered with improved human FcRn resulted in more than 11 h longer half-life.

**Fig. 4:**
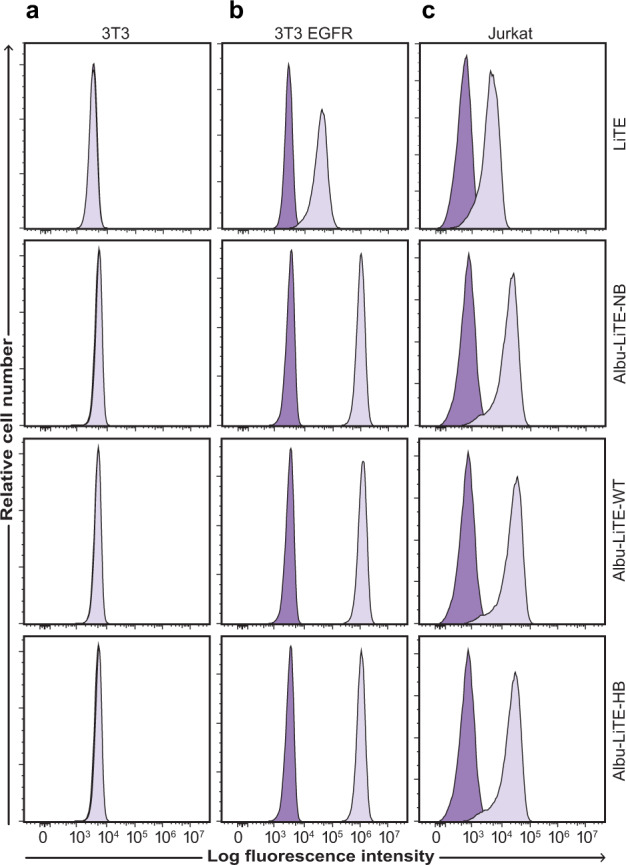
Cell target binding properties of Albu-LiTE constructs. Flow cytometry analyses showing target-specific binding with 5 µg ml^-1^ of the LiTE (102 nM) and Albu-LiTE constructs (46 nM). The LiTE was detected by a C-terminal His-tag directed antibody, while the Albu-LiTE variants were detected using an anti-human serum albumin antibody. **a** No shift in fluorescence intensity was observed for the double-negative 3T3 cell line, whereas, shifts in fluorescence intensities were seen in **b** and **c**. **b** EGFR positive/CD3 negative 3T3 EGFR cells. **c** CD3 positive/ EGFR negative Jurkat T-cells. Isotype control antibody was used for background intensity detection.

Binding of designed Albu-LiTE constructs to EGFR and CD3 was confirmed by flow cytometry using double-negative 3T3 cells, 3T3 EGFR and CD3 expressing Jurkat cell lines (Supplementary Fig. 5). A clear shift in fluorescent intensities was seen for all constructs validating that both the anti-EGFR and anti-CD3 fragments of the LiTE were active with no unspecific binding observed for the double-negative 3T3 cell line (Fig. 4). Notably, the shift in fluorescent intensities was not identical between the conventional LiTE and the Albu-LiTE constructs possibly due to the different detection antibodies and the concentrations used.

**Fig. 5:**
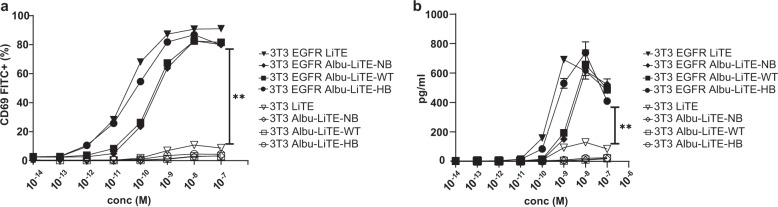
T-cell activation by LiTE and Albu-LiTE constructs. **a** An increasing dose of the tested construct (LiTE; triangle, Albu-LiTE-NB; diamond, Albu-LiTE-WT; square, Albu-LiTE-HB; circle) was added to Jurkat T-cells co-cultured with either 3T3 (open symbols) or 3T3 EGFR cells (closed symbols). The expression of CD69 on the T-cells was evaluated by flow cytometry. **b** Media harvested from the co-cultures were tested for IL-2 secretion in ELISA. Significant differences were observed for T-cell activation on 3T3 or 3T3 EGFR cells in both experiments. Statistical analysis was made by GraphPad prism v.8 using two-way ANOVA with multiple comparisons and Tukey’s post hoc correction, **p < 0.01. Error bars represent standard deviations.

To investigate the EGFR-specific T-cell engagement, Jurkat T-cells were incubated with either EGFR positive (3T3 EGFR) or negative cells (3T3) along with increasing concentrations of the three Albu-LiTE constructs or the LiTE (Fig. 5a). Activation of the Jurkat T-cells was monitored by upregulation of CD69 expression by flow cytometry. The results showed a strong T-cell activation dependent on the concentration of protein constructs and EGFR expression on target cells.

Further validation of T-cell activation was demonstrated by secretion of IL-2 in the conditioned media (Fig. 5b). Similar results to the CD69 upregulation were observed, with increasing IL-2 levels that correlated with increased concentration of LiTE and Albu-LiTE in the samples, but only when EGFR-expressing cells were present. This supports specific and concentration-dependent activation of Jurkat T-cells by LiTE and Albu-LiTE constructs.

**Fig. 6:**
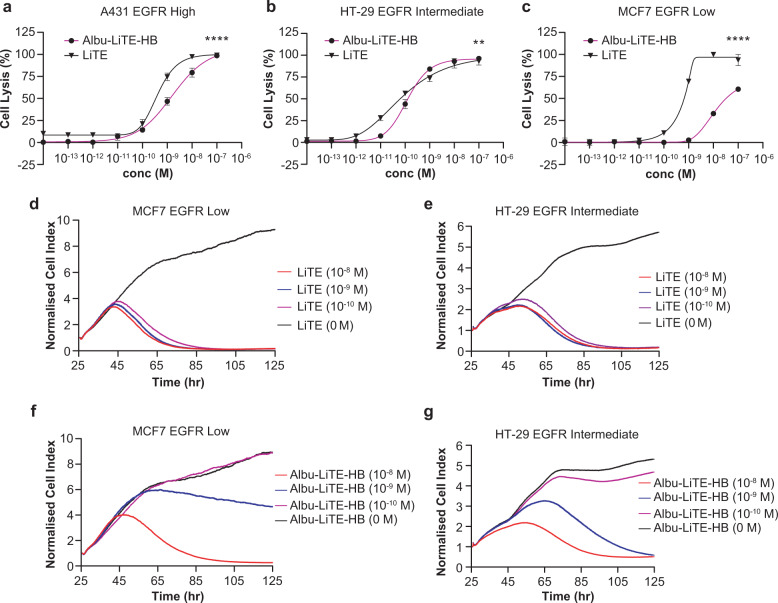
Lactate dehydrogenase (LDH) and real-time cytotoxicity assays of bispecific T-cell-activated cell killing. The effect of increasing concentration of the LiTE (triangle) versus the Albu-LiTE-HB (circle) construct was evaluated using an LDH endpoint assay (**a**–**c**). **a** T-cell-mediated cell killing of high EGFR expressing A431 cells with increasing protein concentrations. **b** T-cell-mediated cell killing of intermediate EGFR expressing HT-29 cells with increasing protein concentrations. **c** T-cell-mediated cell killing of low EGFR expressing MCF7 cells with increasing protein concentrations. For real-time assessment of cell killing, target cells were grown for 25 h and LiTE or Albu-LiTE-HB (10^−8^ M; red line, 10^−9^ M; blue line or 10^−10^ M; purple line) were added together with human peripheral blood mononuclear cells (PBMCs) and the cell index measured every 10 min to monitor cell density (**d**–**g**). **d** Effect on MCF7 EGFR low cell density mixed with PBMCs and various concentrations of LiTE from 24 h after addition compared to the negative control (0 M; black line). **e** Effect on HT-29 EGFR intermediate cell density mixed with PBMCs and various concentrations of LiTE from 24 h after addition compared to the negative control (0 M; black line). **f** Effect on MCF7 EGFR low cell density mixed with PBMCs and various concentrations of Albu-LiTE from 24 h after addition to the negative control (0 M; black line). **g** Effect on HT-29 EGFR intermediate cell density with various concentrations of Albu-LiTE-HB from 24 h after addition compared to the negative control (0 M; black line). Statistical analysis was made in GraphPad prism using two-way ANOVA,**p < 0.005, ****p < 0.0001. Error bars represent standard deviations.

Three different cancer cell lines A431, HT-29 and MCF-7 were evaluated for EGFR expression by flow cytometry and found to be high, medium and low EGFR expressing cells, respectively (Supplementary Fig. 6). The effect of EGFR expression on antibody-mediated T-cell cytotoxicity was investigated using an LDH endpoint assay using freshly isolated human PBMCs as effector cells (Fig. 6a–c).

The killing of tumour cells was EGFR-specific and did not occur in the absence of EGFR expression (Supplementary Fig. 7). Conventional Fc-less LiTE and fusion Albu-LiTE-HB construct-mediated redirected lysis of high and medium EGFR expressing cells with similar potency, however, for the EGFR low MCF-7 a reduced potency was seen of the Albu-LiTE-HB construct (Fig. 6a–c). The mean concentrations for half-maximal lysis (EC50) with MCF7 low EGFR expressing cells were 0.63 nM for LiTE and 31.5 nM for Albu-LiTE-HB. To closer examine this result, a real-time cytotoxicity assay using the xCelligence platform was performed with HT-29 (EGFR medium) cells, MCF7 (EGFR low) cells (Fig. 6d–g) and CHO (EGFR negative) cells (Supplementary Fig. 7). The data confirmed the stronger dose-dependent profile of the Albu-LiTE fusion protein compared to the LiTE molecule. The effect between LiTE and Albu-LiTE-HB could reflect the contribution of the albumin component on immune synapse formation between the T-cells and the target cell lines, as the formation of this very narrow interface could be affected by the larger size of the Albu-LiTE-HB construct including the albumin. The data indicates that albumin containing Albu-LiTE-HB was more dependent on the expression level of EGFR on the target cell, possibly due to less efficient formation of immune synapse.

LiTE and Albu-LiTE constructs were tested for efficacy in a C57BL/6 *RAG1* KO animal model. The knockout of *RAG1* results in a mouse model devoid of T and B lymphocytes. The HT-29 colorectal cancer cell line, which has previously been shown to be resistant to EGFR signalling blockade by cetuximab, due to a downstream mutation in *BRAF*^25^, was used to avoid therapeutic effect of cetuximab by EGFR blockade. This *BRAF* mutation also counters any therapeutic effect from the EgA1 anti-EGFR component in the LiTE that also blocks EGFR signalling and tumour growth^29^. This setup was used to exclude any effect of EGFR blockade on the outcome, and to show the broad applicability of the EGFR-bispecific strategy. The HT-29 cells were inoculated along with human PBMC effector cells at the start of the experiment. Multiple dosing (8 doses) with protein drugs in the model showed superior tumour growth retardation with the LiTE and Albu-LiTE-HB compared to the cetuximab group (Supplementary Fig. 8).

**Fig. 7:**
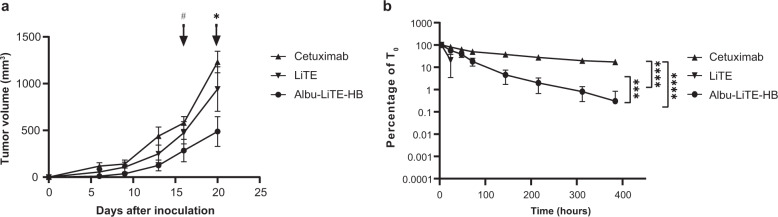
Growth inhibition of HT-29 tumours in AlbuMus *RAG1* KO mice and serum detection of protein drugs. **a** HT-29 cells mixed with human PBMCs were inoculated subcutaneously in immunocompromised AlbuMus *RAG1* KO mice. Animals were divided into three groups (*N* = 6) and injected with cetuximab, LiTE or Albu-LiTE-HB at day 0. Tumour growth was monitored and measured by calliper every 3–4 days. A two-way ANOVA with Holm-Sidak post hoc analysis was used to compare tumour volume between the three groups. Time points where the Albu-Lite-HB tumour group is comparably smaller have been marked *p < 0.01, #*p* < 0.05. Error bars represent SEM. **b** Blood samples were drawn at the 9 time points (1 h, 4 h, 24 h, 48 h, 72 h, 144 h, 216 h, 312 h, 384 h). Full size construct was detected in serum by sandwich ELISA. The LiTE molecule was not detectable in samples drawn after 24 h. The highest concentration in the serum was seen in the 4-hour sample that were set as *T*_0_. Statistical differences between the constructs, detected in serum over time, were analysed using a two-way ANOVA with multiple comparisons and Tukey’s post hoc correction, ****p < 0.0001, ***p = 0.001. Error bars represent standard deviations. All statistical analyses were made in GraphPad prism v. 8.

To fully investigate the efficacy of the albumin fusion constructs we used a new immunocompromised AlbuMus *RAG1* KO model introduced in this work. The absence of T and B-cells in the strain was validated by flow cytometry of freshly isolated splenic and thymic cells (Supplementary Fig. 9). Growth of the human HT-29 colorectal cancer cell line was evaluated after subcutaneous inoculation in AlbuMus *RAG1* KO and compared to the C57BL/6 *RAG1* KO strain (Supplementary Fig. 10). All animals were shown to be susceptible to tumour growth with comparable growth curves between the mouse strains that promotes the application of the new mouse for efficacy studies. Drug efficacy after a single intraperitoneal injection was evaluated in the AlbuMus *RAG1* KO mice. The results showed greater tumour growth retardation of the tumours for the Albu-LiTE-HB fusion compared to the cetuximab and LiTE groups (Fig. 7a). With the single time-point dosing there was no significant effect of the LiTE compared to the cetuximab group, while Albu-LiTE-HB had a significant lower average tumour size at termination of the experiment. ELISA determination of serum levels showed LiTE was rapidly cleared from the blood while Albu-LiTE-HB and cetuximab were clearly detectable in serum samples at all time points (Fig. 7b).

## Discussion

Controlled T-cell redirection is a potential lethal weapon against cancer but comes with the cost of non-specific overstimulation of the immune system observed with bispecific T-cell engager therapies^3,4,8^. Circumvention by removal or silencing of the Fc fragment are the standard strategies employed in all ongoing clinical trials with anti-CD3 bispecific antibody constructs^2^. Removal of the Fc, however, comes at the expense of short half-life and requirement for frequent dosing. Inclusion of an albumin-binding nanobody in drug constructs is a promising strategy that has shown encouraging half-life extension results^15,17^. Recently, investigations into the possible control of drug pharmacokinetics has also been pursued using a pH-sensitive nanobody^30^, with a decreased albumin affinity at acidic endosomal pH. However, only decreased half-life extension was reported compared to other albumin-binding nanobodies. Although inclusion of an albumin-binding domain is promising, it does not allow for the engineering of specific traits into the albumin, as it is dependent on the characteristics of endogenous albumin. Furthermore, the binding is susceptible to competition from endogenous and exogenous ligands^31^.

Bispecific fusion to wild type HSA has previously been shown to increase the half-life of a bispecific single-chain diabody 2½ fold in wild-type mice^32^, although, no target engagement or efficacy data was presented in the work. Discovery of human albumin variants engineered with a set of different FcRn affinities^20^ translatable to modulation of PK^23^, offers a tool-set to programme the PK of drugs based on the albumin sequence built into the drug design. This approach has been applied to BiTE-like molecules in this work and validated in a physiologically relevant AlbuMus model^27^. Furthermore, the introduction of a first immunocompromised version of the AlbuMus allows accurate anti-tumour investigations of an anti-EGFR x anti-CD3 bispecific albumin fusion in *BRAF*-mutated tumours non-respondent to standard anti-EGFR monoclonal therapies.

An Fc-less bispecific T-cell engager LiTE was produced alone, or in fusion to HSA variants (Albu-LiTE-WT, Albu-LiTE-NB or Albu-LiTE-HB) (Supplementary Figs. 1 and 2). Despite a small background signal from the LiTE molecule at pH 5.5 with FcRn in BLI, the expected effect of engineering the different HSA variants into the bispecific design was exhibited (Fig. 2, Supplementary Fig. 3). The three Albu-LiTE constructs exhibited expected FcRn binding and FcRn-driven in vitro cellular recycling characteristics (Fig. 2 and Table 1). Previous work from our group has shown that the level of in vitro FcRn-engagement correlates with circulatory half-life of a N-terminal fusion of protein fragments to HSA variants^22^. In the present work, we investigated the circulatory half-life of the bispecific HSA constructs in an *hFcRn*^+/+^, *hAlb*^+/+^ AlbuMus model^27^. The endogenous HSA pool is crucial for detecting the full effect of altered FcRn engagement on the PK of HSA designs as it avoids the competition from endogenous mouse albumin that exhibits higher affinity for mouse and human FcRn^33^. The data revealed rapid clearance of the Fc-less LiTE molecule (*t*_½_ = 0.59 h) that was undetectable in blood samples taken 48 h post injection. The circulatory half-life of Albu-LiTE constructs was programmable by the HSA sequence built into the design demonstrated by an incremental increase that corresponded to FcRn affinity. The Albu-LiTE-NB showed shortest half-life (*t*_½_ = 18.68 h), but still considerably elevated above unfused LiTE that most likely reflects reduction in renal clearance due to increase in molecular weight of the construct (Fig. 3). The Albu-LiTE-WT and Albu-LiTE-HB were detectable at 9 days post injection with the Albu-LiTE-HB showing superior half-life extension compared to wild type (*t*_½_ = 37.20 h and 25.90 h, respectively). This half-life extension technology offers longer-lasting effects with less frequent dose requirements and concomitant lower side-effects and better patient compliance than conventional Fc-less bispecific formats. The ability to program the half-life of molecules by the albumin sequences has great potential for improving the therapeutic window of highly potent and hard-to-control drugs like T-cell engaging bispecific antibodies. The complicated nature and cross-species differences make it difficult to fully predict human response to drugs based on surrogate animal models; the programmable half-life platform introduced in this work may provide an additional level of control to obtain maximum efficacy with minimal adverse effects when moving into clinical trials.

In situ binding of cellular antigens was shown by flow cytometry for all Albu-LiTE constructs (Fig. 4). All constructs initiated specific T-cell activation in vitro in the presence of EGFR-positive target cells, but not the EGFR-negative counterpart, shown by the upregulation of CD69 and IL-2 secretion (Fig. 5a, b). The results indicate that the unfused LiTE was more potent to elicit T-cell activation than the Albu-LiTE fusions. This was further investigated using an LDH endpoint cytotoxicity assay on cancer cell lines displaying a varying degree of EGFR expression (Fig. 6a–c and Supplementary Fig. 6). A more potent T-cell-mediated cell killing from the LiTE compared to Albu-LiTE-HB was shown. Previous reports have shown T-cell-mediated cytotoxicity is highly sensitive to even small changes on the immune synapse formation by bispecific T-cell engagers^34,35^ and it is likely that albumin could cause interference in the efficiency of immune synapse formation or signalling^32^, although its potential impact on the functionality of binding domains also cannot be ruled out. The results, however, suggest much stronger selective cytotoxicity based on EGFR expression levels with the Albu-LiTE-HB, which was further corroborated by real-time measurements of drug-induced T-cell-mediated cell killing (Fig. 6d–g). Correlation between induction of T-cell cytotoxicity by anti-CD3 bispecific T-cell engagers and increased expression level of antigens on target cells has previously been shown and associated with epitope location and the size of the extracellular domain of the targeted tumour-associated antigen^34,36^. Our data suggests that Albu-LiTE-HB can discriminate between cells with different levels of antigen expression, potentially providing a strategy for targeting antigens that are highly expressed by cancer cells but have lower expression in normal tissues, such as EGFR. Such discrimination in cell killing may lead to reduction in on-target off-tumour toxicity in healthy tissue displaying lower levels of the target antigen, leading to an increased therapeutic window similar to affinity tuning explored for CAR-T cells^37^. Initial screening of the in vivo activity of the LiTE and Albu-LiTE-HB was investigated in an immunodeficient C57BL/6 *RAG1* knockout mouse model inoculated with a 2:1 mixture of HT-29 cells and human PBMCs using multiple (8) drug doses to compensate for the short half-life of the unfused LiTE (Supplementary Fig. 8). Anti-tumour efficacy was compared to the anti-EGFR antibody cetuximab to exclude any EGFR blockade effects on tumour growth inhibition^25^ and determine the ability of a bispecific format to inhibit *BRAF*-mutated tumours by EGFR-specific T-cell activation. Tumour growth was significantly inhibited in both the LiTE and Albu-LiTE-HB cohorts compared to the cetuximab cohort. In order to observe the true effect of albumin half-life extension on tumour growth we have developed the first immunodeficient version of the AlbuMus model. This was performed by CRISPR/Cas9 knockout of the *RAG1* gene resulting in the absence of CD3^+^ T-cells and CD19^+^ B-cells (Supplementary Fig. 9) in a model with the appropriate *hFcRn*^+/+^, *hAlb*^+/+^ phenotype. This strain was susceptible to human HT-29 tumour growth comparable to the isogenic C57BL/6 *RAG1* KO strain (Supplementary Fig. 10) that allowed efficacy studies to be performed. This AlbuMus *RAG1* KO may in the future become the gold-standard animal model for evaluation of oncology drugs fused to HSA or any oncology drugs that interact with the endogenous albumin pool. A single dosing regime was employed to exemplify the benefit of increased half-life of the albumin fusion on efficacy and the presence of injected protein was detected in serum samples (Fig. 7b). Tumour growth inhibition was markedly greater for the half-life extended Albu-LiTE-HB compared to the LiTE molecule following a single dose (Fig. 7a) in spite of the higher potency shown with the LiTE in vitro shown with the HT-29 cells in the real-time cytotoxicity assay (Fig. 6e, g). Detection of Albu-LiTE-HB in the serum throughout the experiment indicates that the advantage over LiTE is coupled to enhanced circulation time, and the lack of efficacy in the cetuximab group further indicates the effect to be due to EGFR-specific T-cell activation. The fading of the observed initial growth inhibition is likely due to the lack of additional T-cell co-stimulation, the limited lifespan of the co-inoculated PBMCs and the absence of T-cell influx into the developing tumours, as would normally occur in an immunocompetent host. While T-cell redirection by bispecific antibodies has proven extremely potent, it has also been reported that this initial stimulation over time leads to an exhausted phenotype in the T-cell population causing the anti-tumour effect to subside. Critical co-stimulation of T-cells or blockade of checkpoint inhibitor signalling by antibodies as a combinatorial treatment may prove to be crucial to get a complete and lasting response. Beneficial inhibition of the PD-1/PD-L1 axis in combination with CD3 containing bispecific antibodies has been shown for leukaemia^36,38^ and multiple myeloma^34^ and will undoubtedly play a similar role for solid tumours.

Albumin exhibits wide tissue distribution^39^ and passive tumour accumulation^40^ with albumin drugs reported to enter by receptor-mediated processes in a caveolae-dependent manner^41^. Recent work from our laboratory has identified FcRn overexpression in human cancer and higher tumour accumulation in mice of an FcRn high-binding recombinant albumin variant^42^ promoting adoption for targeted albumin-drug fusion designs. Furthermore, the ability of endogenous albumin to disseminate into the lymph system has been exploited for T-cell stimulation in tumour-associated lymph nodes^43,44^. This suggests that BiTE-like albumin fusions could be used to target primary and disseminated disease and should be the focus of future work.

In this work we have engineered a panel of BiTE-like albumin fusions with extended circulatory half-life programmed by the FcRn affinity of the albumin sequence that exhibit improved anti-tumour effects in a beyond state-of-the-art animal model. An approach that could offer optimised therapeutic effects of BiTE-like drugs without the requirement for frequent dosing and with a lower risk of adverse events arising from Fc interactions with the immune system.

## Methods

### Cell lines and culture conditions

HEK293E (ATCC, CRL-10852) and Jurkat cells (ATCC, TIB-152) were cultured in RPMI media (Gibco, #61870-010) supplemented with 10% fetal bovine serum (FBS) (Sigma, #10500-064) and 1% PenStrep (Sigma, #15140-122). HT-29 colorectal cancer cells (ATCC, HTB-38) were grown in McCoys 5 A basal media (Gibco) with 10% FBS and 1% PenStrep. A431 epidermoid carcinoma cells (ATCC, CRL-1555) were grown in DMEM (Gibco, #41966-029) with 10% FBS and 1% PenStrep. MCF7 breast adenocarcinoma cells (ATCC, HTB-22) were grown in DMEM (Gibco) with 10% FBS and 1% PenStrep. CHO Chinese hamster ovary cells (ATCC, CCL-61) were grown in DMEM with 10% FBS and 1% PenStrep. 3T3 and 3T3-EGFR cells lines were cultured in DMEM media with 10% FBS and 1% PenStrep as previously described^26^. HMEC1-FcRn cells (previously generated in collaboration with Novozymes)^18^ were cultured in MCDB131 medium (Thermo Fisher Scientific, #10372019) supplemented with 10% FBS, 2 mM L-glutamine, 10 ng ml^−1^ human EGF (Peprotech), 1 ng ml^−1^ fibroblast growth factor (Peprotech), 50 µg ml^−1^ gentamicin (Sigma, #G1397) and 0.25 µg ml^−1^ Fungizone (FisherScientific, #15290018). All cell lines were cultured in humidified atmosphere at 37°C with 5% CO_2_.

### cDNA design and construction of expression vectors

The LiTE^26^ cDNA construct was synthesised (Genscript) with unique restriction sites between each protein domain i.e. the EgA1 nanobody, the OKT3 scFv and subcloned into vectors containing wild-type HSA and variants with high FcRn binding affinity (HB) or removed FcRn binding affinity (NB)^45^. The vectors further contained CMV enhancer and promoter supporting expression in HEK293E cells. For the LiTE protein a C-terminal His-tag was included for purification and detection purposes; this was omitted in the Albu-LiTE fusion to avoid interference with FcRn binding.

### Protein expression and purification

HSA fusion proteins were produced by transient transfection of HEK293E cells. Transfected cells were grown in serum-free 293Freestyle media (Gibco, #12338018). Secreted protein was purified from supernatant using CaptureSelect Human albumin affinity matrix (ThermoFisher, #191297005) by elution with 2 M MgCl_2_, pH 7.4. Purified protein was concentrated and buffer exchanged into PBS by Vivaspin2 centrifugal concentrators (Sartorius). The bispecific LiTE protein was expressed and purified by Nickel-NTA followed by protein A affinity chromatography, as described elsewhere^26^. For size exclusion chromatography the protein sample in 100 mM Tris-Hcl pH 7.4 was run using a Yarra™ 1.8 µm SEC-X150 column (Phenomenex) on a Dionex Ultimate 3000 HPLC (ThermoFisher Scientific) with a flowrate of 0.1 mL per minute. A protein standard ranging from 670 kDa to 0.244 kDa (Phenomenex) was run with the same settings and overlaid for comparison.

### SDS-PAGE and Western blot analysis

Samples were separated on a 10% SDS-PAGE gel and stained by Coomassie brilliant blue or blotted onto a PVDF membrane. Membranes were blocked in 2% w/v skim milk powder dissolved in PBS (mPBS) and incubated for 4 h with horseradish peroxidase (HRP)-conjugated polyclonal sheep anti-HSA (Abcam, #ab8941) diluted 1:2000 in 2% mPBS and washed 4 times in PBS, before developing with TMB substrate (Sigma, #T0565) for membranes.

### FcRn-binding kinetics using Bio-Layer Interferometry

Binding kinetics of human FcRn for purified Albu-LiTE constructs was measured by Bio-Layer Interferometry (BLI) on an Octet Red96e system (ForteBio/Molecular Devices). Recombinant HSA variants without fusion protein corresponding to WT, no or high FcRn affinity were used as controls^45^. Briefly, biotinylated soluble human-FcRn (Immunitrack, # ITF02) was immobilised on streptavidin-coated biosensors (ForteBio/Molecular Devices) in PBS pH 7.4 supplemented with 0.01% Tween-20 at a 8.75 nM concentration. For kinetic characterisation, a 7-step two-fold dilution series starting at 3 µM was prepared in 25 mM Na-acetate, 25 mM NaH_2_PO_4_, 150 mM NaCl and 0.01% Tween-20 at pH 5.5. Binding kinetics was performed at 30 °C with a 300 s association phase and 600 s dissociation phase. The sensors were regenerated in PBS pH 7.4 supplemented with 0.01% Tween-20 and equilibrated in 25 mM Na-acetate, 25 mM NaH_2_PO_4_, 150 mM NaCl and 0.01% Tween-20 pH 5.5 between samples. All data were referenced with FcRn–streptavidin sensors in buffer without analyte. Data analysis was performed using the Octet data analysis software ver. 10.0 (ForteBio/Molecular Devices) using curve fitting to a 1:1 model for estimation of kinetic parameters.

### FcRn cellular recycling assay

Cellular recycling assay was performed as described by Schimdt et al.^18^. Briefly, HMEC-1 cells, engineered to stably overexpress *hFcRn* (HMEC-1-FcRn), were seeded 1 × 10^5^ cells per well in 48 well plates coated with GelTrex (ThermoFisher, # A1413201). Cells were grown to confluency before experimental use. Cells were washed twice in prewarmed PBS followed by the addition of 0.15 µM Albu-LiTE construct or HSA control (300 µl per well) in Hanks balanced salt solution pre-adjusted to pH 6.0 by 1 M MES buffer. After a 1-hour incubation at 37 °C, 5% CO_2_, the cells were washed 5 times in ice-cold PBS. Then 160 µl per well complete medium without FBS was added and cells were incubated for 1 h to allow recycling and release of internalised protein, supernatants were collected and analysed by sandwich ELISA.

For sandwich ELISA, Maxisorp plates were coated overnight at 4 °C with a polyclonal goat anti-HSA antibody (Sigma, # A-7544) diluted 1:1000 in PBS for the unfused HSAs or with monoclonal rabbit anti-camelid VHH antibody (Genscript, # A01860) diluted 1:1000 in PBS for the Albu-LiTE fusion proteins. Coated plates were blocked for 1 h with 2% casein blocking buffer (Sigma, #C7594-1L) before washing with PBS + 0.05% Tween and loading of the construct samples and albumin standard. Samples were incubated for 1 h followed by washing and incubation with an HRP-conjugated secondary polyclonal sheep anti-HSA antibody (Abcam, #ab8941). All samples were run in triplicates. Data was analysed on GraphPad Prism v. 8 using Mann–Whitney test.

### Validation of cellular antigen recognition by flow cytometry

For each sample, 1 × 10^6^ Jurkat, 3T3, 3T3 EGFR, A431, HT-29, MCF7 or CHO cells were washed in ice-cold assay buffer (PBS + 1% FBS) and incubated on ice with 5 µg ml^−1^ of LiTE (102 nM), Albu-LiTE construct (46 nM), recombinant HSA (75 nM) (Sigma, #A6608), human anti-EGFR cetuximab (33 nM) (MerckSerono, # 090232), mouse anti-human CD3ε-FITC (33 nM) (ImmunoTools, # 21850033) or anti-mouse IgG2a isotype control FITC (33 nM) (ImmunoTools, #21225023) in 100 µl assay buffer for 1 h. Cells were pelleted by centrifugation and washed in ice-cold assay buffer. Secondary antibodies, anti-rabbit Alexa488 (Invitrogen, #A11034) and anti-human IgG Alexa488 (Invitrogen, #A11013) were added to appropriate samples and incubated on ice for 30 min. Unfused LiTE construct was detected by mouse anti-His antibody (Sigma, #A7058) followed by anti-mouse Alexa488 (Invitrogen, # A32723). Albu-LiTE constructs were detected by rabbit anti-HSA antibody (ThermoFisher, # MA5-29022) followed by anti-rabbit Alexa488 (Invitrogen, # A32731) secondary antibody. Cells were pelleted by centrifugation and washed in ice-cold assay buffer. All samples were resuspended in 7-ADD (Invitrogen, #A1310) diluted 1:100 in assay buffer for live/dead staining. Samples were run on a Novocyte flow cytometer (ACEA Biosc Inc.) using 488 and 561 nm excitation and 530/30 and 660/20 filter sets. Data was analysed using FlowJo version 10 software (FlowJo, LLC).

### T-cell activation and CD69 upregulation

Target cells and effector Jurkat cells were seeded into Nunc™ 96-well plates (Nunc, # 168136) at an effector to target cell (E:T) ratio of 5:1. The 3T3 cell line was used as negative control while EGFR-expressing 3T3 EGFR stable transfected cells were used as the positive control. Antibody test samples diluted in assay media (supplemented RPMI-1640 Glutamax medium; Gibco, # 41966-029) were added into appropriate wells. After an overnight co-culture period at 37°C, the plates were centrifuged and cell supernatants were collected for ELISA analysis. The effector cells were harvested and stained with anti-CD69 FITC antibody, diluted 1:100 (Biolegend, #104506), and anti-HLA class I APC antibody, diluted 1:100 (Biolegend, # 311410) for 30 min at 4°C. Cells were washed twice in PBS + 1% FBS and resuspended in 200 μL of 7-AAD viability dye diluted 1:200 (Invitrogen, #A1310). Samples were run on a Novocyte flow cytometer (ACEA Biosc Inc.). Data was analysed using FlowJo version 10 software (FlowJo, LLC).

### ELISA detection of human Interleukin-2 secretion

Detection of secreted human Interleukin 2 (hIL-2) was made by a hIL-2 kit (Diaclone, # 950.010.096) according to company protocol. Briefly, Maxisorp 96-well plates (Nunc, cat# 442404) were coated with 100 μL of capture antibody and incubated overnight at 4°C. The wells were washed twice with wash buffer (PBS + 0.05% Tween20). 100 μL of blocking buffer (PBS + 5% BSA) was added to the wells and incubated for 2 h at room temperature (RT). 100 μL of IL-2 standards and cell supernatant harvested from T-cell activation assay were added into appropriate wells followed by addition of 50 μL of biotinylated detection antibody. After 1-hour incubation at RT, washing was performed and 100 μL of Streptavidin-HRP was added and incubated at RT for 30 min. All wells were washed 4 times and 100 μL of TMB-substrate was added. Samples were incubated in the dark for 5–15 min at RT. 100 μL of 1 M sulfuric acid was added to quench the reaction. Absorbance was measured at 450 nm (630 nm as reference wavelength) on a Clariostar plate reader (BMG Labtech). Data was analysed in GraphPad Prism v. 8.

### PBMC isolation from whole blood

Human blood sample was collected from a healthy donor in EDTA-coated Vacutainer tubes (Becton Dickson, #367525). Blood sample was diluted 1:1 with PBS. Equal volume of diluted blood was carefully layered over Ficoll-Paque PLUS solution (GE Healthcare, #17144002) in a 50 mL Falcon tube. The tubes were centrifuged with acceleration and brakes turned off, at 400 *g* 20 °C for 30 min. The upper layer of the resulting gradient containing plasma and platelets was removed using a sterile pipette. The buffy coat containing the peripheral blood mononuclear cells (PBMCs) was carefully isolated using a pipette. The PBMCs were treated with ACK lysing buffer (Gibco, #A1049201) to remove any red blood cells. The final PBMCs were washed in PBS and resuspended in assay medium.

### Lactate dehydrogenase (LDH) cellular toxicity assay

Target cells were seeded overnight into Nunc™ 96-well plates (Nunc, #161093). A431, HT-29 and MCF7 cells lines, which express the targeted antigen EGFR, were used as positive cell lines while a CHO cell line was used as a negative control cell line. After 24 h of incubation, antibody test samples diluted in assay media (supplemented RPMI-1640 Glutamax medium; Gibco, # 32404-014) and freshly isolated effector cells PBMC (E:T = 5:1) were added into appropriate wells. After 48 h of co-culture, a final concentration of 1% Triton X-100 (Sigma-Aldrich, #T8787) solution was added into high control wells to obtain maximal cell lysis. Target cells incubated with effector PBMC only were used as the low control. The plates were centrifuged and cell supernatants were collected for cytotoxicity analysis using the LDH cytotoxicity detection kit (Takarabio, #MK401) following the manufacturer’s protocol. 100 µL of cell supernatant was added to 100 µL of reaction mixture, which was prepared according to the manual and incubated for 10–15 min at RT. Absorbance was measured at 492 nm (600 nm as reference wavelength) on a Clariostar plate reader (BMG Labtech). Data was analysed in GraphPad Prism v. 8. Cytotoxicity was calculated following the equation, Cytotoxicity (%) = ((Experiment value − Low control) / (High control − Low control)) x 100.

### Cellular toxicity using xCELLigence^®^ Real-time Cell Analysis (RTCA)

Real-time cytotoxic effects of the antibody constructs on cancer cell lines were studied using the xCELLigence^®^ RTCA instrument (ACEA Biosc Inc.) at 37°C and 5% CO_2_. 50 μL of cell culture media (supplemented RPMI-1640 Glutamax medium; Gibco, # 41966-029) was added to all wells on the E-plate 16 plates (ACEA Biosc Inc., # 5469830001) and a short baseline measurement was recorded by the instrument using the RTCA Software 1.0. Target cell lines were then seeded on the E-plates and measurements were recorded every 10 min for 24 h. 50 μL of antibody diluted in cell culture media and 50 μL of freshly isolated effector PBMCs (E:T = 5) were added to appropriate wells and measurements were recorded every 10 min for 96 h. Cell index (CI) values obtained were normalised to the CI values recorded after 24 h of incubation. Data was plotted and analysed using GraphPad Prism v. 8. Cytotoxicity was calculated following the equation: Cytotoxicity (%) = ((Experiment value  − Low control) / (Low control − 1)) x 100, where low control consisted of target cells incubated with effector PBMC only without antibody constructs.

### Animal models

All animal experiments were performed at the Animal Facility, Department of Biomedicine, Aarhus University, under the Danish Animal Experiment Inspectorate license #2018-15-0201-01399 and with ethical approval in accordance with the national guidelines for care and use of laboratory animals. For tumour xenografts a tumour volume of 1200 mm^3^ was set as the humane endpoint.

Pharmacokinetic studies were performed in male and female double transgenic human FcRn (*hFcRn*^+ /+^)/human serum albumin (*hAlb*^+ /+^) “AlbuMus” with a C57BL/6 background^27^.

For tumour growth studies the C57BL/6 with a *RAG1* knockout-*RAG1*^tm1Mom^/J (The Jackson Laboratory, # B6.129S7) was used for comparing standard tumour growth of HT-29 to an AlbuMus *RAG1* KO strain. AlbuMus *RAG1*-edited founder mice were generated by genOway (Lyon, France). A guide RNA (5′ CATGGCAGAATTCCGTCGGG 3′) was designed to specifically cut into *RAG1* exon 2. Premature STOP codons were inserted thanks to the use of a ssODN oligonucleotide. The CRISPR sgRNA was then microinjected into fertilised oocytes obtained from C57Bl/6 homozygous double humanised FcRn/albumin mouse line (genOway proprietary model^27^). DNA from new-born mice was then isolated and characterised by PCR amplification to assess *RAG1* exon-2 sequence. Three founders were identified and bred to obtain heterozygous animals. Both the C57BL/6 *RAG1* KO and the AlbuMus *RAG1* KO strains were used for drug efficacy investigations.

### Pharmacokinetics study

For studying the PK, seven randomly mixed male and female AlbuMus were allocated for each experimental protein group and five to the PBS control group. Mice were injected i.v. through the tail vein with 2 mg kg^−1^ drug or PBS. Blood samples were collected in 20 µl heparinized micro capillary tubes (Marienfeld, #2911110) at 1 min, 1 h, 4 h, 24 h, 48 h, 96 h, 168 h and 216 h. The first blood samples were taken from sub-lingual blood and the subsequent samples from the tail vein. Samples were diluted in PBS, spun down and serum fractions were stored for later construct detection by sandwich ELISA.

For ELISA, Maxisorp plates were coated overnight at 4 °C with monoclonal rabbit anti-camelid V_HH_ antibody (Genscript, #A01860) diluted 1:1000 in PBS. Coated plates were washed once in PBS and blocked for 4 h with 2% mPBS. Blocking buffer was removed, samples diluted in PBS and dilution series of known concentrations were added and incubated overnight at 4 °C. The wells were washed once in PBS + 0.05% Tween-20 and 3 times in PBS, 200 µl per well. Sheep anti-HSA antibody (Abcam, #ab8941) diluted 1:2000 in 2% mPBS was incubated 4 h at RT. For detection of the LiTE construct anti-polyHistidine peroxidase antibody was used 1:1000 diluted in 2% mPBS. The wells were washed 4 times in PBS and developed using TMB PLUS2 (Kem-En-Tec Diagnostics, #4395). Samples were prepared in triplicate. Data was analysed in the Graphpad Prism software v.8 by a two-phase decay model after interpolation to standard series.

### HT-29 colorectal cancer cell growth in mouse models

C57BL/6 *RAG1* KO (*N* = 6) and AlbuMus *RAG1* KO (*N* = 17) animals were used at 8 weeks of age. For each animal 3 × 10^6^ HT-29 cells were mixed 1:1 v/v with LDEV-free extracellular matrix protein Geltrex (Gibco, # A1413201) and inoculated by subcutaneous injection in the right flank. Tumours were measured by calliper every 3–4 days and tumour volume was calculated based on the formula Volume = π/(6 × *L* × *W*)^2^ and geometrical means of normalised and averaged volumes were plotted. Mann–Whitney U-test did not show significant difference between the curves *p* > 0.9999. Statistics were made using Graphpad prism v. 8.

### Tumour growth inhibition in C57BL/6 *RAG1* KO mice and AlbuMus *RAG1* KO

For the drug multiple dosing experiment, 6 female C57BL/6 *RAG1* KO were allocated to each of the groups, cetuximab, LiTE or Albu-LiTE-HB, respectively. Each animal was inoculated by subcutaneous injection of 2 × 10^6^ HT-29 cells mixed with 1 × 10^6^ freshly isolated human PBMCs, cells were mixed 1:1 v/v with LDEV-free extracellular matrix protein Geltrex (Gibco, # A1413201) and inoculated in the right flank under anaesthesia. Proteins were intraperitoneally injected with 5.0 mg kg^−1^ cetuximab (833 pmol per animal), 1.0 mg kg^−1^ Albu-LiTE-HB (230 pmol per animal) or 0.5 mg kg^−1^ LiTE (230 pmol per animal). Animals were dosed a total of 8 times with the first dose at the time of inoculation and another 7 times each time tumours were measured. Tumours were measured by calliper every 3–4 days and tumour volume was calculated based on the formula Volume = π/(6 × *L* × *W*^2^).

For the single drug administration experiment, male and female AlbuMus *RAG1* KO were used with *N* = 6 for each treatment group except for PBS treatment *N* = 3. Each animal was inoculated by subcutaneous injection of 1 × 10^6^ HT-29 cells mixed with 2 × 10^6^ freshly isolated human PBMCs, cells were mixed 1:1 v/v with LDEV-free extracellular matrix protein Geltrex and inoculated in the right flank under anaesthesia. Proteins were injected intraperitoneally at the time of inoculation with 20 mg kg^−1^ cetuximab (3.33 nmol per animal), 2.2 mg kg^−1^ Albu-LiTE construct (500 pmol per animal) or 1.0 mg kg^−1^ LiTE (500 pmol per animal). Tumours were measured by calliper every 3–4 days and tumour volume was calculated based on the formula Volume = π/(6 × *L* × *W*^2^).

Proteins were detected in blood samples taken from the tail vein in 20 µl Heparin coated capillaries at the time of tumour inoculation and at an additional 8 time points (4, 24, 48, 72, 144, 216, 312 and 384 h). Blood samples were analysed in ELISA for protein drug content, as described above.

### Statistics and reproducibility

All data were analysed using GraphPad Prism 8 Software. The specific type of statistical analysis utilised is indicated in the corresponding figure legends. The differences between the groups were evaluated by various statistical tests, which are defined in the methods section and in individual figure legends. Exact *p*-values are indicated in figures. A minimum value of *p* < 0.05 was considered to be statistically significant. All in vitro experiments were repeated at least two times to ensure reproducibility.

### Reporting summary

Further information on research design is available in the Nature Research Reporting Summary linked to this article.

## Supplementary information

Supplementary Information

Description of Additional Supplementary Files

Supplementary Data 1

Reporting Summary

## Data Availability

All data are available from the corresponding author upon reasonable request. Source data underlying graphs and charts presented figures can be found in Supplementary Data 1.
