# Supplementary material for: Programmable half-life and anti-tumour effects of bispecific T-cell engager-albumin fusions with tuned FcRn affinity
Source: Commun Biol. 2021 Mar 8;4:310. doi: 10.1038/s42003-021-01790-2 (PMC7940400; doi:10.1038/s42003-021-01790-2)
Supplement: Supplementary file 1 — Supplementary Information [file 42003_2021_1790_MOESM1_ESM.pdf]

Programmable Half-life and Anti-Tumour Effects of Bispecific T-Cell Engager-Albumin Fusions with Tuned FcRn Affinity

Ole A. Mandrup[1], Sui Ching Ong[1], Simon Lykkemark[1], Anders Dinesen[1], Imke Rudnik-Jansen[1], Niels Frederik Dagnæs-Hansen[2], Jan Terje Andersen[3,4], Luis Alvarez-Vallina[5,6] and Kenneth A. Howard[1*]

[1]Interdisciplinary Nanoscience Center (iNANO), Department of Molecular Biology and Genetics, Aarhus University, DK-8000 Aarhus C, Denmark.

[2]Department of Biomedicine, Aarhus University, DK-8000 Aarhus C, Denmark.

[3]Department of Immunology, University of Oslo, Oslo University Hospital Rikshospitalet, Oslo, Norway.

[4]Institute of Clinical Medicine and Department of Pharmacology, University of Oslo, Oslo, Norway.

[5]Cancer Immunotherapy Unit (UNICA), Department of Immunology, Hospital Universitario 12 de Octubre, Madrid, Spain.

[6]Immuno-Oncology and Immunotherapy Group, Instituto de Investigación Sanitaria 12 de Octubre (i+mas12), Madrid, Spain.

*Corresponding author; kenh@inano.au.dk.

Supplementary Information

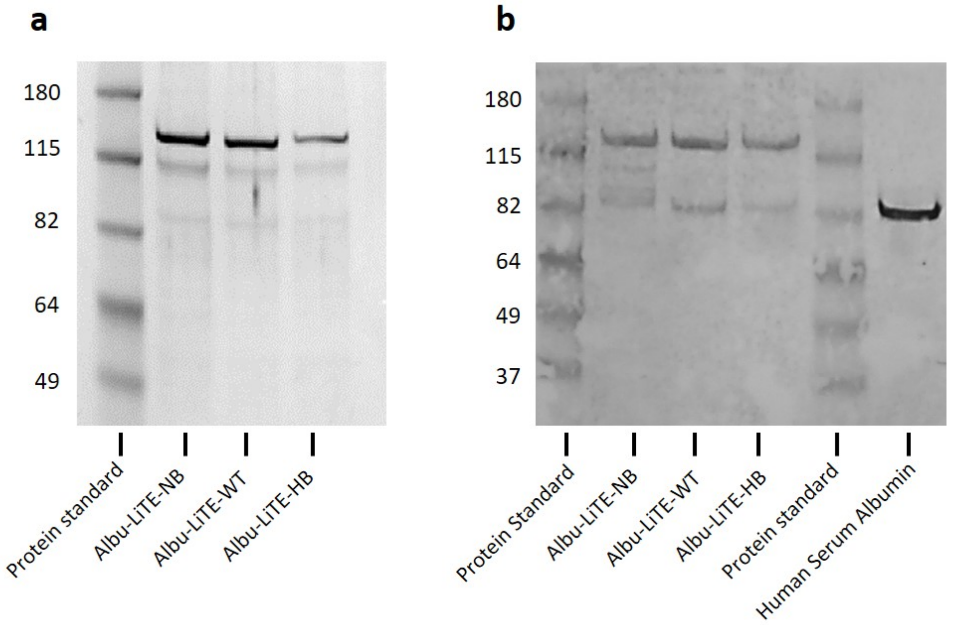

Supplementary Figure 1. **SDS-PAGE and Western blot analysis of expressed protein**. **a**. Coomassie blue staining showing the purity of Albu-LiTE construct on SDS-PAGE after purification. **b**. Western blot analysis using anti-human albumin antibody for validation of purified protein. Human serum albumin was included for size comparison.

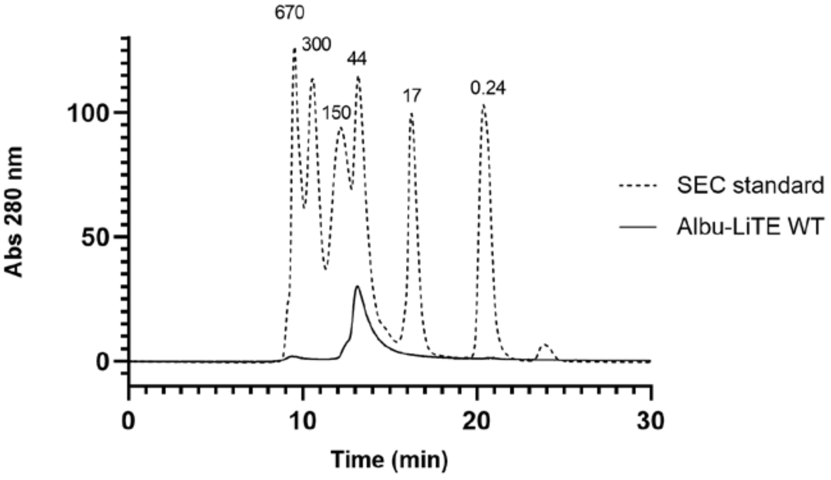

Supplementary Figure 2. **Size exclusion chromatography of purified Albu-LiTE protein**. Purified Albu-LiTE construct was run on HPLC using a size exclusion chromatography column. A size exclusion protein standard (SEC) was run for size estimation.

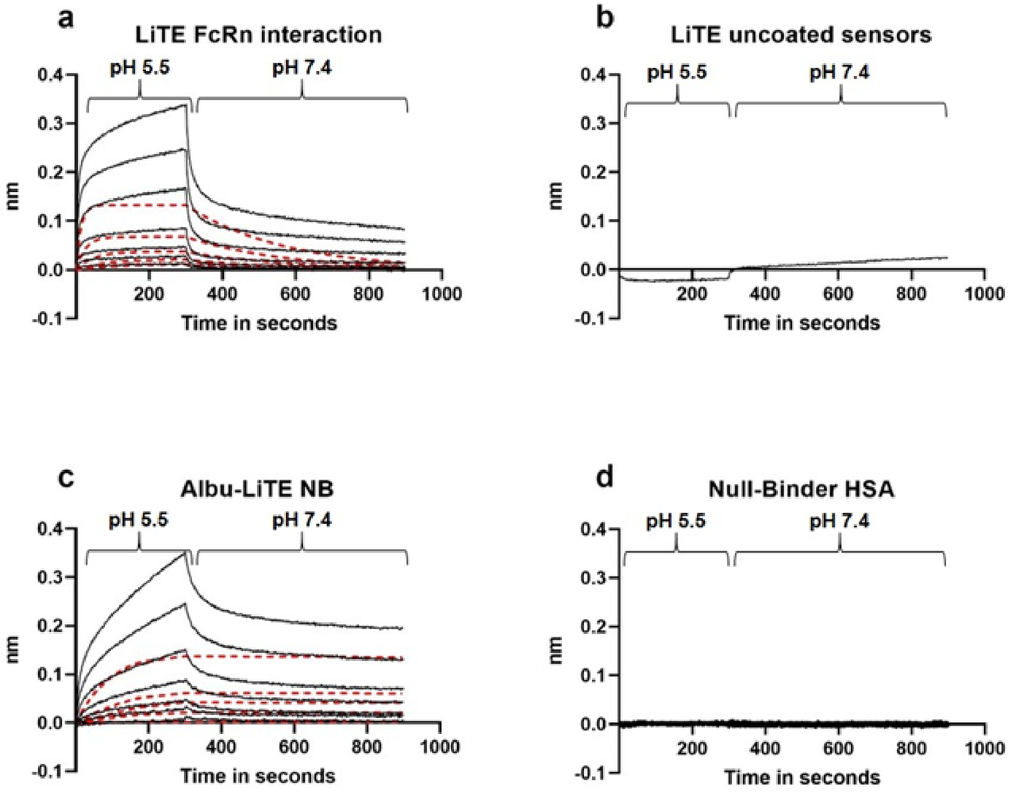

Supplementary Figure 3. **Kinetic measurements**. Sensorgrams showing protein binding initially at pH 5.5 followed by pH 7.4. **a**. Binding curves of serial dilutions with LiTE on sensor tips with immobilized human FcRn. **b**. Binding curve of LiTE on un-coated sensor tips. **c**. Binding curves of serial dilutions with Albu-LiTE NB on sensor tips with immobilized human FcRn. **d**. Binding curves of serial dilutions with Null-Binder HSA on sensor tips with immobilized human FcRn. Failed fitting with a 1:1 model of sensorgrams are shown by red dotted lines.

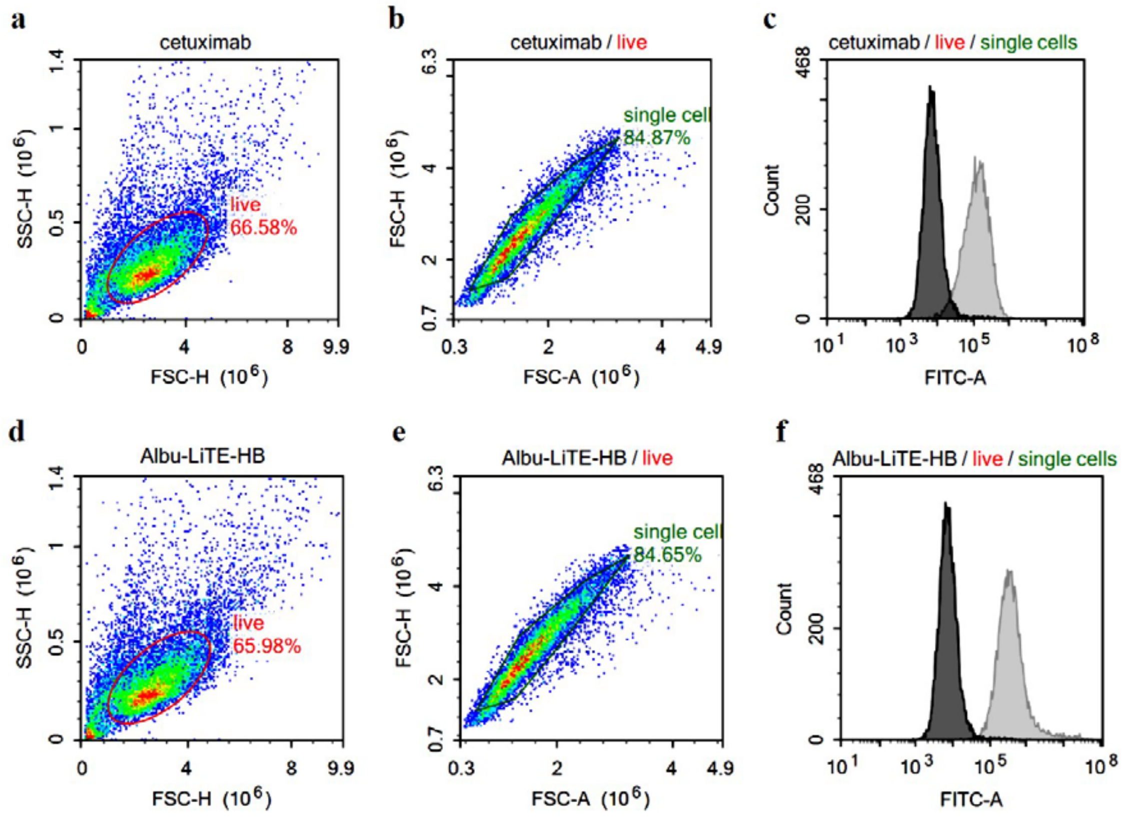

Supplementary Figure 4. **EGFR expression in the HMEC-1 cell line.** The expression of EGFR was investigated by flow cytometry. **a**. Gating for live cell population. **b**. Gating for single cells among the live population **c**. The anti-EGFR antibody cetuximab was incubated with HMEC-1 cells and fluorescence measured in the live single cell population. **d**. Gating for live cell population. **e**. Gating for single cells among the live population **f**. The Albu-LiTE-HB construct containing the anti-EGFR nanobody EgA1 was incubated with HMEC-1 cells and fluorescence measured in the live single cell population. Dark grey: signal from isotype control antibody, Light grey signal from FITC conjugated secondary antibody against EGFR binding protein.

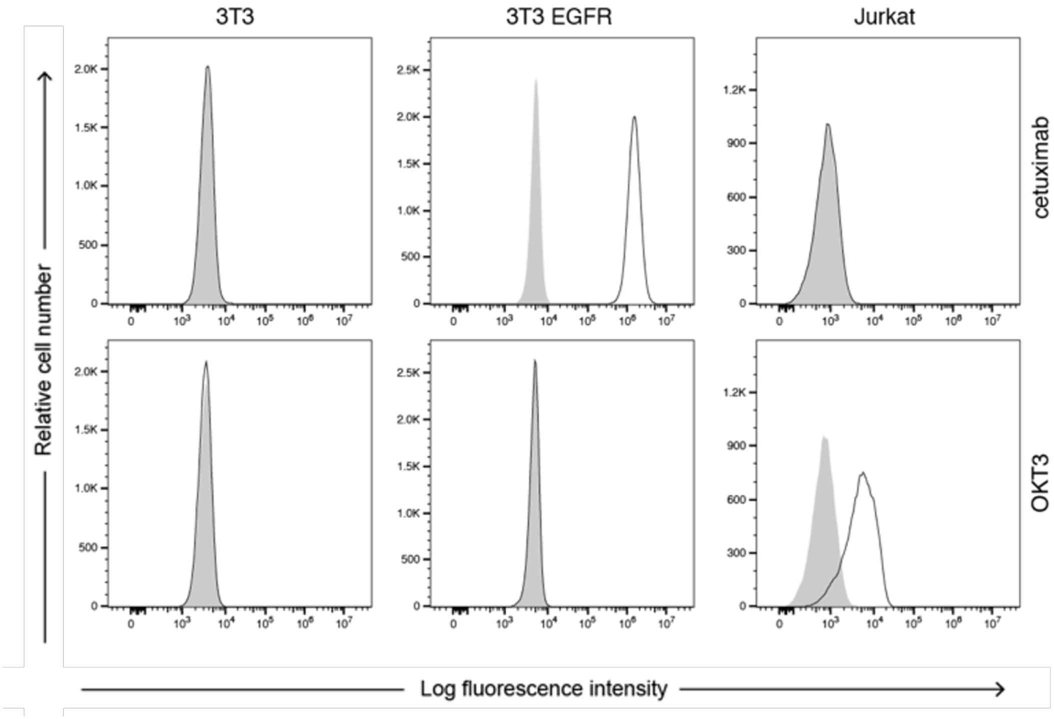

Supplementary Figure 5. **Validation of antigen expression.** Expression of EGFR and CD3 was investigated by cetuximab anti-EGFR and OKT3 anti-CD3 antibodies. Dark grey represent isotype control antibody and black lines represent fluorescent shift for cells bound by cetuximab (top) and OKT3 (bottom) antibodies respectively.

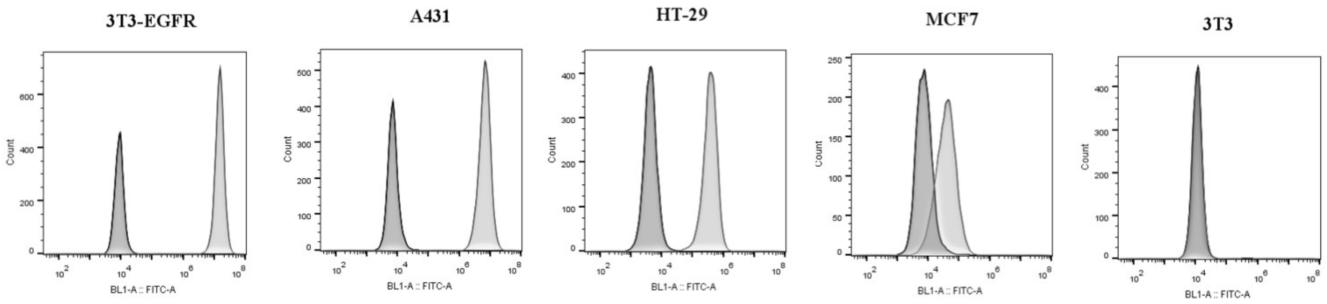

Supplementary Figure 6. **Evaluation of EGFR expression in cell lines.** The expression of EGFR was evaluated by binding of cetuximab in flow cytometry for A431, HT-29 and MCF-7 cancer cell lines. The expression levels are seen in relation to the very high EGFR expressing 3T3 EGFR cell line and the EGFR negative 3T3 cell line. Shifts in fluorescence with cetuximab binding (light grey) is compared to unstained cells (dark grey).

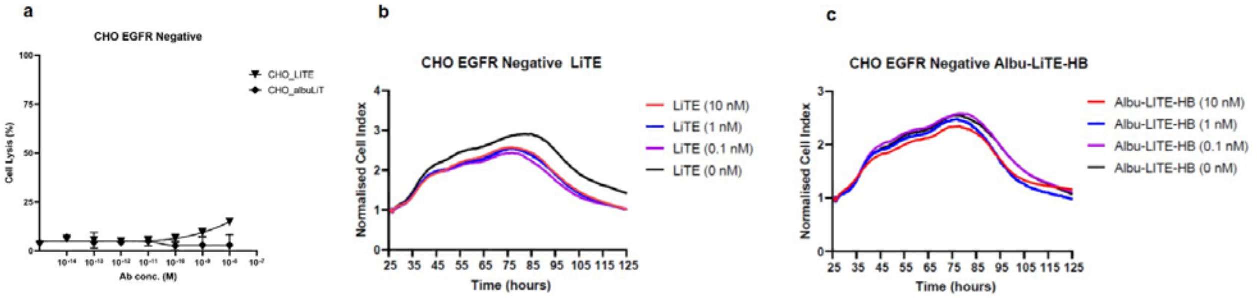

Supplementary Figure 7. **T-cell activated cell killing on EGFR negative CHO cells.** As a control for background level of cell killing hEGFR negative CHO cells were grown and incubated with freshly isolated human PBMCs and protein constructs. **a.** increasing concentrations of either LiTE (triangles) or Albu-LiTE (diamonds) was added to the cells. **b.** CHO **c**ells were grown for 25 hours and LiTE or **c.** Albu-LiTE-HB (10, 1 or 0.1 nM) was added along with human PBMCs, the cell index was measured every 10 minutes to track cell density. Error bars represent standard deviations.

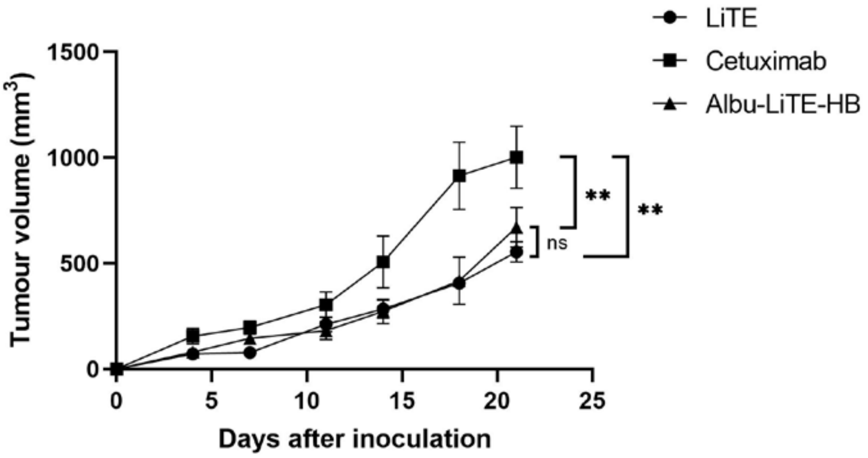

Supplementary Figure 8. **Tumour growth inhibition of HT-29 cells in C57BL/6 *RAG1* KO.** A mixture of HT-29 cells with human PBMCs (2:1) were inoculated s.c. in immunocompromised C57BL/6 *RAG1* KO mice. Animals were divided into three groups (N=6) and injected with either cetuximab (squares), LiTE (circles) or Albu-LiTE-HB (triangles) at day 0, 4, 7, 11, 14, 18 and 21. Tumour growth was monitored and measured by callipers every 3-4 days. Statistical analysis was made in GraphPad prism using two-way ANOVA with *post-hoc* correction for multiple comparison, ** = $p < 0.01$, n.s. = not significant. Error bars represent S.E.M.

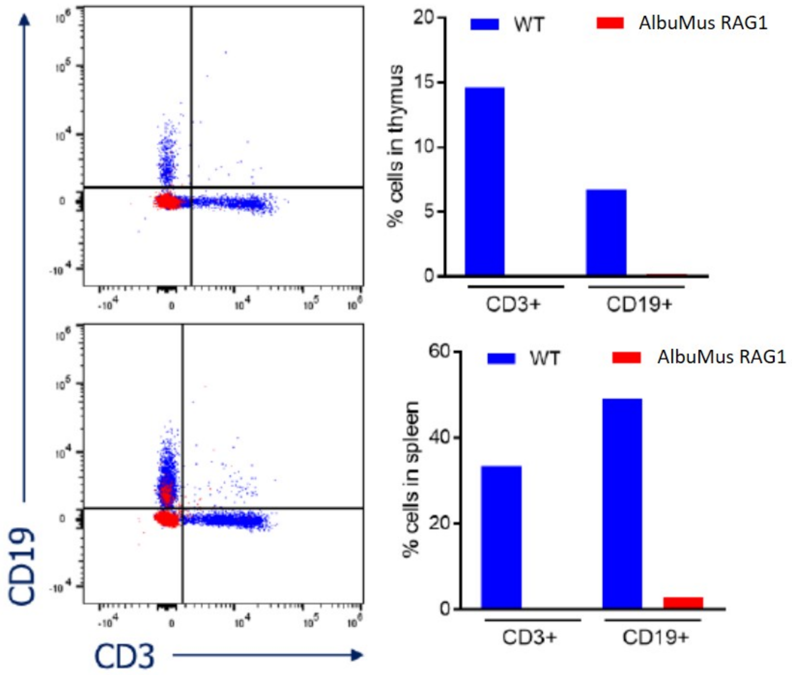

Supplementary Figure 9. **Flow cytometric analysis for CD3 positive T-cells and CD19 positive B-cells in an AlbuMus *RAG1* KO mouse**. Freshly isolated splenocyte and thymocytes from AlbuMus *RAG1* KO mice were analysed for presence of B- and T-cells and compared to numbers found in wild-type mice (WT).

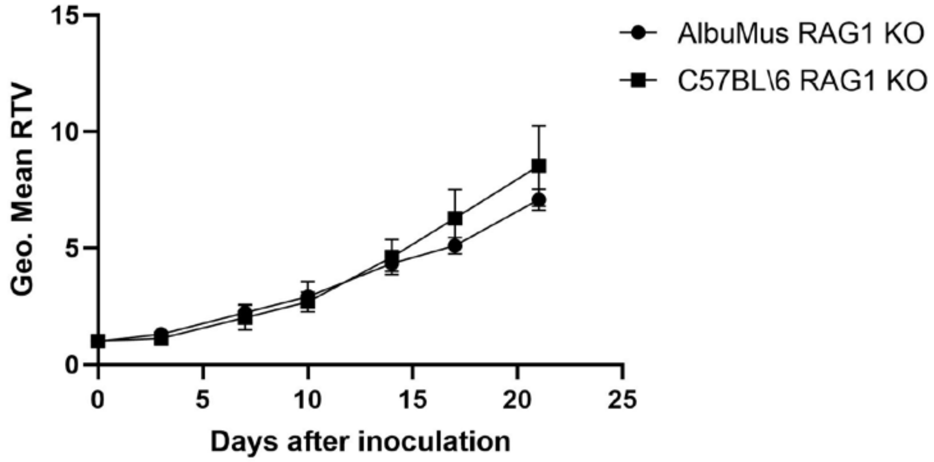

Supplementary Figure 10. **Tumour growth characterisation in an AlbuMus *RAG1* KO.** HT-29 cells were inoculated subcutaneous in the right flank of AlbuMus *RAG1* KO (N=17) and compared to the growth of tumour likewise inoculated in C57BL\6 *RAG1* KO mouse (N=6). The geometric mean of relative tumour volumes was calculated and plotted in the graph. Statistical analysis was made in GraphPad prism and tumour growth was not statistically different between the two mouse models. Error bars represent standard deviations.
