# Supplementary material for: Programmable half-life and anti-tumour effects of bispecific T-cell engager-albumin fusions with tuned FcRn affinity
Source: Commun Biol. 2021 Mar 8;4:310. doi: 10.1038/s42003-021-01790-2 (PMC7940400; doi:10.1038/s42003-021-01790-2)
Supplement: Supplementary file 2 — Description of Additional Supplementary Files [file 42003_2021_1790_MOESM2_ESM.pdf]

Description of Additional Supplementary Files

File name: Supplementary Data 1

Description: Source data underlying graphs and charts.
