# Supplementary material for: Programmable half-life and anti-tumour effects of bispecific T-cell engager-albumin fusions with tuned FcRn affinity
Source: Commun Biol. 2021 Mar 8;4:310. doi: 10.1038/s42003-021-01790-2 (PMC7940400; doi:10.1038/s42003-021-01790-2)
Supplement: Supplementary file 4 — Reporting Summary [file 42003_2021_1790_MOESM4_ESM.pdf]

nature research

Corresponding author(s): Kenneth A. Howard

Last updated by author(s): Jan 20, 2021

Reporting Summary

Nature Research wishes to improve the reproducibility of the work that we publish. This form provides structure for consistency and transparency in reporting. For further information on Nature Research policies, see our Editorial Policies and the Editorial Policy Checklist.

Statistics

For all statistical analyses, confirm that the following items are present in the figure legend, table legend, main text, or Methods section.

n/a	Confirmed	
☐	☒	The exact sample size (*n*) for each experimental group/condition, given as a discrete number and unit of measurement
☐	☒	A statement on whether measurements were taken from distinct samples or whether the same sample was measured repeatedly
☐	☒	The statistical test(s) used AND whether they are one- or two-sided<br>*Only common tests should be described solely by name; describe more complex techniques in the Methods section.*
☒	☐	A description of all covariates tested
☐	☒	A description of any assumptions or corrections, such as tests of normality and adjustment for multiple comparisons
☐	☒	A full description of the statistical parameters including central tendency (e.g. means) or other basic estimates (e.g. regression coefficient) AND variation (e.g. standard deviation) or associated estimates of uncertainty (e.g. confidence intervals)
☐	☒	For null hypothesis testing, the test statistic (e.g. F, t, r) with confidence intervals, effect sizes, degrees of freedom and P value noted<br>*Give P values as exact values whenever suitable.*
☒	☐	For Bayesian analysis, information on the choice of priors and Markov chain Monte Carlo settings
☒	☐	For hierarchical and complex designs, identification of the appropriate level for tests and full reporting of outcomes
☒	☐	Estimates of effect sizes (e.g. Cohen's d, Pearson's r), indicating how they were calculated

Our web collection on statistics for biologists contains articles on many of the points above.

Software and code

Policy information about availability of computer code

Data collection	For Octet BLI data collection, Octet system software v.10 (Forte Bio) was used
Data analysis	Generally for analysis Graphpad Prism software v. 8 was used.<br>For analysis of Octet binding curves, Octet system software, Octet Analysis v.10 (Forte Bio) was used and analysed data was transfered to Graphpad Prism for visualisation.<br>For flow cytometry data FlowJo v. 10 was used

For manuscripts utilizing custom algorithms or software that are central to the research but not yet described in published literature, software must be made available to editors and reviewers. We strongly encourage code deposition in a community repository (e.g. GitHub). See the Nature Research guidelines for submitting code & software for further information.

Data

Policy information about availability of data

All manuscripts must include a data availability statement. This statement should provide the following information, where applicable:

- Accession codes, unique identifiers, or web links for publicly available datasets
- A list of figures that have associated raw data
- A description of any restrictions on data availability

Supporting data have been included as a Supplementary Data file. The data that support the findings of this study are further available from the corresponding author upon reasonable request

Field-specific reporting

Please select the one below that is the best fit for your research. If you are not sure, read the appropriate sections before making your selection.

☒ Life sciences ☐ Behavioural & social sciences ☐ Ecological, evolutionary & environmental sciences

For a reference copy of the document with all sections, see nature.com/documents/nr-reporting-summary-flat.pdf

Life sciences study design

All studies must disclose on these points even when the disclosure is negative.

Sample size	Sample size was chosen based on a previously published studies (Viuff et al., Journal of controlled release 223 (2016) 22–30; Schelde et al., J. Biol. Chem.(2019) 294(10) 3735–374; Lutterbuese et al. PNAS 2010 vol 107 (12605–12610))
Data exclusions	Data was not excluded from the analysis
Replication	All in vitro experiments were reproduced at least once. Replications of animal experiments have not been performed
Randomization	For in vivio studies animals were allocated to the groups so that animals of each sex would be equally distributed. Animals were otherwise randomly distributed
Blinding	For in vivo tumour measurments technicians collecting the data was blinded

Reporting for specific materials, systems and methods

We require information from authors about some types of materials, experimental systems and methods used in many studies. Here, indicate whether each material, system or method listed is relevant to your study. If you are not sure if a list item applies to your research, read the appropriate section before selecting a response.

Materials & experimental systems

n/a	Involved in the study
☐	☒ Antibodies
☐	☒ Eukaryotic cell lines
☒	☐ Palaeontology and archaeology
☐	☒ Animals and other organisms
☒	☐ Human research participants
☒	☐ Clinical data
☒	☐ Dual use research of concern

Methods

n/a	Involved in the study
☒	☐ ChIP-seq
☐	☒ Flow cytometry
☒	☐ MRI-based neuroimaging

Antibodies

Antibodies used	human anti-EGFR cetuximab (MerckSerono, # 090232), mouse anti-human CD3ε-FITC (ImmunoTools, # 21850033), anti-mouse IgG2a isotype control FITC (ImmunoTools, #21225023), anti-rabbit Alexa488 (Invitrogen, #A11034), anti-human IgG Alexa488 (Invitrogen, #A11013), anti-mouse Alexa488 (Invitrogen, # A32723), mouse anti-His antibody (Sigma, #A7058), rabbit anti-HSA antibody (ThermoFisher, # MA5-29022), anti-rabbit Alexa488 (Invitrogen, # A32731), anti-CD69 FITC antibody (Biolegend, #104506), anti-HLA class I APC antibody (Biolegend, # 311410), monoclonal rabbit anti-camelid VHH antibody (Genscript, #A01860), Sheep anti-HSA antibody (Abcam, #ab8941), goat anti-HSA antibody (Sigma, # A-7544), polyclonal sheep anti-HSA antibody (Abcam, #ab8941)
Validation	Only antibodies validated for the specific assay and species reactivity by the supplier were used

Eukaryotic cell lines

Policy information about cell lines

Cell line source(s)	All cell lines were purchased from ATCC, except for the HMEC-1-FcRn which was accessible through collaborations (Manuscript Ref 13, Schmidt et al.), and 3T3 EGFR cells which was available through collaborations (Manuscript ref 20, Molgaard et al.)
Authentication	Cell lines authenticated by ATCC
Mycoplasma contamination	Cell lines were tested negative for contamination with mycoplasm by PCR
Commonly misidentified lines (See ICLAC register)	non

Animals and other organisms

Policy information about studies involving animals; ARRIVE guidelines recommended for reporting animal research

Laboratory animals	Female C57BL/6 with a RAG1 knockout-RAG1tm1Mom/J (The Jackson Laboratory, # B6.129S7). Male and female double transgenic human FcRn (hFcRn +/+)/human serum albumin (hAlb +/+) "AlbuMus" with a C57BL/6 background. Male and female immunocompromised RAG1 knockout, double transgenic human FcRn (hFcRn +/+)/human serum albumin (hAlb +/+) "AlbuMus" with a C57BL/6 background
Wild animals	The study did not make use of any wild animals
Field-collected samples	The study did not use material collected from field
Ethics oversight	The study was made in accordance with national guidelines and under a ethical approval by national authorities. Danish Animal Experiment Inspectorate license #2018-15-0201-01399

Note that full information on the approval of the study protocol must also be provided in the manuscript.

Flow Cytometry

Plots

Confirm that:

- [ ] The axis labels state the marker and fluorochrome used (e.g. CD4-FITC).
- [x] The axis scales are clearly visible. Include numbers along axes only for bottom left plot of group (a 'group' is an analysis of identical markers).
- [x] All plots are contour plots with outliers or pseudocolor plots.
- [x] A numerical value for number of cells or percentage (with statistics) is provided.

Methodology

Sample preparation	Sample were prepared from cultured cell lines. Cells growing attached to a plastic surface was released by trypsination and thoroughly washed to remove neutralised trypsin
Instrument	Novocyte flow cytometer (ACEA Biosc Inc.)
Software	Data was collected using NovoExpress software and analysed using FlowJo v.10
Cell population abundance	Flowcytometry data was made with cultured cell lines and number of cells determined by automated cell counter. Experiments was made with 10000 cells per sample
Gating strategy	Gating were used in some experiments to gate for live single cells. Representative gatings are shown in Supplementary Figure 4

- [x] Tick this box to confirm that a figure exemplifying the gating strategy is provided in the Supplementary Information.
